# Exendin-4 Exacerbates Burn-Induced Morbidity in Mice by Activation of the Sympathetic Nervous System

**DOI:** 10.1155/2019/2750528

**Published:** 2019-01-17

**Authors:** Xiao-Jing Ji, Ji-Wei Hao, Guang-Lei Li, Ning Dong, Xin-Qi Wang, Min Zhou, Qing-Hong Zhang, Yong-Ming Yao

**Affiliations:** ^1^Trauma Research Center, Fourth Medical Center of Chinese PLA General Hospital, Beijing 100048, China; ^2^Neurocritical Care Unit, First Affiliated Hospital of USTC, Division of Life Sciences and Medicine, University of Science and Technology of China, Hefei, Anhui 230001, China

## Abstract

**Background:**

Although glucagon-like peptide 1- (GLP-1-) based therapy of hyperglycemia in burn injury has shown great potential in clinical trials, its safety is seldom evaluated. We hypothesize that exendin-4, a GLP-1 analogue, might affect the immune response via the activation of the sympathetic nervous system in burn injury.

**Methods:**

Male Balb/c mice were subjected to sham or thermal injury of 15% total body surface area. Exendin-4 on T cell function *in vitro* was examined in cultured splenocytes in the presence of *β*-adrenoceptor antagonist propranolol (1 nmol/L) or GLP-1R antagonist exendin (9-39) (1 *μ*mol/L), whereas its *in vivo* effect was determined by i.p. injection of exendin-4 (2.4 nmol/kg) in mice. To further elucidate the sympathetic mechanism, propranolol (30 mg/kg) or vehicle was applied 30 min prior to injury.

**Results:**

Although the exacerbated burn-induced mortality by exendin-4 was worsened by propranolol pretreatment, the inhibition of T cell proliferation by exendin-4 *in vitro* could be restored by propranolol instead of exendin (9-39). However, a Th2 switch by exendin-4 *in vitro* could only be reversed by exendin (9-39). Likewise, the inhibition of splenic T cell function and NFAT activity by exendin-4 *in vivo* was restored by propranolol. By contrast, the increased splenic NF-*κ*B translocation by exendin-4 *in vivo* was potentiated by propranolol in sham mice but suppressed in burn mice. Accordingly, propranolol abrogated the heightened inflammatory response in the lung and the accelerated organ injuries by exendin-4 in burn mice. On the contrary, a Th2 switch and higher serum levels of inflammatory mediators by exendin-4 were potentiated by propranolol in burn mice. Lastly, exendin-4 raised serum stress hormones which could be remarkably augmented by propranolol.

**Conclusions:**

Exendin-4 suppresses T cell function and promotes organ inflammation through the activation of the sympathetic nervous system, while elicits Th2 switch via GLP-1R in burn injury.

## 1. Introduction

Incretin-based therapies including glucagon-like peptide 1 (GLP-1) and its analogues in the management of acute hyperglycemia with improved glucose tolerance in critical illness [1–6], e.g., burn injury [7–9], have shown great potential in clinical trials. However, the evaluation of its safety concerns in critically care especially the inflammatory response is extremely limited [10]. Accumulating studies, albeit controversial, have suggested that GLP-1 be associated with inflammatory response. Although reduced plasma GLP-1 level was found in burn-injured rats [9], increased serum GLP-1 secretion was observed in patients with acute and chronic inflammatory processes such as sepsis [11], chronic kidney disease [11], as well as in mice under inflammatory stimuli [12]. Actually, GLP-1 receptor (GLP-1R) mRNA transcripts were detected in the lymphocytes from nonobese diabetic (NOD) mice [13], in the invariant natural killer T cells (iNKT) from the psoriasis patients [14], and in the macrophages from normal mice [15]. Furthermore, it has been suggested that GLP-1 exerts an inhibitory effect on the immune system. In adaptive immunity, GLP-1 analogue exendin-4 (Ex-4) could increase the number of CD4^+^CD8^+^ T cells in the lymph nodes [13] and pancreas [16], but could inhibit the migration of human CD4^+^ T cells [17]. Moreover, it could reduce the frequency of regulatory T cells (Tregs) in the thymus [13, 18], but it could increase in the spleen with a trend of enhanced suppression ability [19]. Ex-4 therapy could decrease the frequency of iNKT cells in psoriasis patients [14], but could activate them in diabetic patients and obese mice [20]. The immunosuppressive effect of Ex-4 was also demonstrated by its reduction of cytokine production from intestinal intraepithelial lymphocyte in mice [21, 22] and from peripheral blood mononuclear cells in diabetic patients [23]. In innate immunity, Ex-4 confers an anti-inflammatory effect [22] by increasing IL-10 expression in the peripheral blood and pancreas [16]. Meanwhile, it downregulated TNF-*α* and monocyte chemoattractant protein-1 (MCP-1) in macrophages [15] and in the airway of asthma mice [24] through inactivation of nuclear factor kappa B (NF-*κ*B) [15]. Additionally, it reduced monocyte/macrophage accumulation in the arterial wall [15] and adipocytes [25] in mice.

Furthermore, as a neuropeptide released from both the brain and the intestine, GLP-1 could activate the sympathetic nervous system (SNS) both peripherally and centrally. In human, infusion of GLP-1 significantly increased sympathetic activity systemically [26, 27] or locally in muscle [28]. In rats, acute administration of high doses of Ex-4 either peripherally or centrally could also elicit a SNS response with an increased urine level of epinephrine metabolite [29]. Notably, central GLP-1R activation could increase the expression of dopamine-*β*-hydroxylase, a key enzyme in noradrenaline synthesis in the brain of mice [30, 31], and significantly increase sympathetic nerve activity in the peripheral lipid in mice [32]. However, plasma catecholamine was suppressed substantially in diabetic individuals after a 2-week treatment with dipeptidyl peptidase-4 inhibitor, which significantly raised plasma GLP-1 level [33], indicating that the effect of GLP-1 on the SNS was an acute event.

Increasing evidence suggests that the SNS be involved in the modulation of the cellular immune system via adrenoceptor-dependent mechanism [34, 35]. It was reported that T cell activation be modulated by norepinephrine [36], and the abnormality of a subset of CD4^+^ T cell was associated with SNS hyperactivity [37]. In an animal model of stroke, an extensive apoptotic loss of lymphocytes and Th2 shift was mediated by catecholamine [38]. Moreover, targeting the *β*-adrenoceptor (*β*-AR) on lymphocytes leads to inhibition of inflammatory cytokine production, lymphocyte killing [39], and CD4^+^ T lymphocyte proliferation [40]. Intriguingly, ample evidence showed that trigging *β*2-ARs could intersect with the NF-*κ*B signaling cascade at different levels in a cell type and gene-specific manner [41]. Regarding the inflammatory response, SNS activation induced by stress or injury led to monocytic IL-10 release [42], IL-6 production in normal skin [43], myelopoiesis [44], and chemotaxis of monocytes [45]. Propranolol (Prop) is a nonselective *β*-AR antagonist that mitigates the catecholamine response associated with burns. However, results on the immunomodulatory role of *β*-blockade from burn injury [46, 47] and sepsis [48, 49] are mixed.

Although GLP-1 and its analogues have shown great potential in managing hyperglycemia in critical illness, literature remains sparse as to the immunomodulatory property of Ex-4 in critical illness. Likewise, few studies have passed out the indirect effect of SNS stimulation by Ex-4 versus direct effect via GLP-1R-cAMP stimulation on immune response. We thereby pursued the GLP-1 action on the immune response and the underlying SNS-dependent mechanism in thermal-injured mice. We now demonstrate that T cell function, organ inflammation, and organ injury are controlled by exogenous Ex-4 in a SNS-dependent manner, whereas T cell differentiation and systemic inflammation are promoted by Ex-4 via GLP-1R activation. Elucidation of its potential mechanism for immunoregulation of GLP-1R agonist in critical illness would help to avoid the side effect of incretin-based therapy [50].

## 2. Materials and Methods

### 2.1. Animals and Thermal Injury Model

Male Balb/c mice (20-25 g, Huafukang Bioscience Co. Inc., Beijing, China) were acclimatized for one week before exposed to 94°C thermal injury for 8 seconds of 15% total body surface area (TBSA) after anesthetized with diethyl ether as previously reported [51]. Sham-injured mice were subjected to all of the procedures except the bath that was 37°C. Mice received 1.0 mL Ringer's solution subcutaneously immediately after injury for fluid resuscitation. A topical antibacterial agent (iodine tincture) was applied on the wound. The mice were caged individually with warm bedding in a temperature and humidity controlled room with 12 h light and 12 h darkness. Food and water were provided ad libitum. Animals were sacrificed, between 9:00 a.m. and 10:00 a.m. by cervical dislocation. Analgesics were not used owning to the profound immunosuppressive effects of opiates. All experimental manipulations were undertaken in accordance with the National Institutes of Health *Guide for the Care and Use of Laboratory Animals*, with the approval of the Scientific Investigation Board of the Chinese PLA General Hospital, Beijing, China.

### 2.2. Experimental Designs

For ex vivo study, aliquots of 4 × 10^5^ splenocytes separated from both sham and burn mice 24 h following injury were cultivated in the presence of T cell mitogen concanavalin A (5 mg/L; ConA, Sigma, MD, USA) in 96-well plates containing 200 *μ*L/well of complete RMPI 1640 medium at 37°C with a humidified 5% CO_2_/air atmosphere. Consecutive concentrations of Ex-4 (E7144; 0.3-30 nmol/L, Sigma) [15] were added into the medium with or without propranolol hydrochloride (1 nmol/L; Prop, P8688, Sigma) [42] or exendin (9-39) (1 *μ*mol/L; Ex-9, E7269, Sigma, MD, USA) pretreatment for 1 h [21].

For *in vivo* study, the burn mice were separated into four subgroups: vehicle, Ex-4, Ex-4 plus Prop, and Prop (*n* = 25 − 28/subgroup). Peripheral adrenoceptor blockade with Prop (30 mg/kg) [52] or vehicle (sterile 0.01 M PBS) was commenced intraperitoneally (i.p.) 30 min before injury. Then, 15%TBSA thermal injury was carried out followed by i.p. injection of Ex-4 (2.4 nmol/kg, Sigma) [53] or vehicle 30 min after injury [15, 54]. Thereafter, animals were left in home cages with water and food freely, and the survival rates were documented at 4 h intervals until 7 days. In another set of experiment, both the sham and burn mice were separated into four subgroups as above (*n* = 20/subgroup) and underwent the same procedures. They were killed at 6 h and 24 h (*n* = 6 − 8/group/time point), respectively, and after injury, blood and tissues were collected as follows.

### 2.3. Blood and Tissue Collection

Blood was drained from the posterior orbital vein of ether-anesthetized mice into the tubes containing with or without 10 *μ*L heparin at 6 h and 24 h after injury. The samples were centrifuged at 1000*g* for 10 min at 4°C. In the meantime, another investigator simultaneously removed the spleen and lung. All tissue samples were flash frozen on liquid nitrogen and stored at -80°C.

### 2.4. Separation of Splenocyte and Purification of T Cells

The spleens were harvested 24 h after injury and put into ice-cold RPMI1640 culture media. Splenocyte suspensions were obtained by flushing through 70 *μ*m mesh (BD Biosciences, Beijing, China) with a syringe and were purified in lysis buffer (Applygen Technologies Inc.) to remove the red cells. Cell suspensions were then washed and resuspended in complete culture medium (RPMI 1640 supplemented with 10% heat-inactivated fetal bovine serum). Cells were then allowed to adhere on a plastic Petri dishes (10 mm in diameter, BD Biosciences) at 5 × 10^6^ cells/mL for 2 h to remove the adherent myeloid cells at 37°C in a 5% CO_2_. Nonadherent cells, mainly the lymphocytes, were resuspended in complete culture medium and used in subsequent studies [40, 55, 56]. To determine the purity of nonadherent lymphocytes, 1 × 10^6^ separated cells in staining buffer (1% BSA in 0.01 M PBS) were incubated with PE-anti-mouse CD3*ε* (2.5 *μ*g/100 *μ*L, cat. 100307, BioLegend, CA, USA) for 30 min on ice. Cells were washed with 2 mL staining buffer and centrifuged at 1500 rpm for 5 min at 4°C before analyzed on a FACScan cytometer (BD, Franklin Lakes, New Jersey, USA).

### 2.5. Immunofluorescence of GLP-1R on T Cells

Sections with purified CD3^+^ T cells were fixed in 4% polyformaldehyde for 20 min and were blocked in 3% bovine serum albumin (BSA, Sigma) for 1 h at room temperature and stained overnight at 4°C with GLP-1R antibody (1 : 100; sc-66911, Santa Cruz, USA) and rat anti-mouse CD3 (1 : 100; eBioscience, USA) in PBS including 1% BSA. After washed 3 times with PBS, sections were incubated in DyLight™ 549-conjugated AffiniPure goat anti-rabbit and anti-mouse IgG (H+L) antibodies (1 : 200; Jackson ImmunoResearch Laboratories, West Grove, USA) for 2 h at room temperature in dark box. Slides were rinsed and coverslips were affixed with VECTASHIELD mounting medium containing 4′,6-diamidino-2-phenylindole (DAPI) for nucleus staining (Vector Laboratories, Burlingame, CA, USA). Control experiments were performed with unspecific isotype antibodies of rabbit instead of the primary antibody. Fluorescent cell images were obtained on a confocal laser scanning microscope (LSM 700, Carl Zeiss MicroImaging, Jena, Germany).

### 2.6. Analysis of T Cell Function and Differentiation

Splenocytes were cultured in complete medium stimulated by ConA (5 mg/L, Sigma) for 48 h. Cell-free supernatant fractions were collected and stored at -80°C until cytokines analysis by ELISA (ExCell Biology Inc., Shanghai, China). T cell differentiation was represented by Th1 cytokine secretion of IFN*γ* and Th2 cytokine secretion of IL-4. T cell function was represented by cell proliferation using a CCK-8 method (Dojindo Molecular Technologies Inc., Shanghai, China) in a multiplate spectrophotometer (Spectra MR; Dynex, Richfield, MN, USA) and IL-2 secretion by ELISA (ExCell Biology Inc.).

### 2.7. Measurement of NFATc1 Activation in T Cells and NF-*κ*B Activation in Splenocytes

The calcineurin/nuclear factor of activated T cells (NFATc1) is activated upon T cell receptor activation followed by intracytoplasmatic calcium influx [57] and subsequent nuclear translocation [58]. Nuclear extracts from purified lymphocytes of both groups at 6 h and 24 h postburn were prepared using a nuclear extraction kit as described (Applygen Technologies Inc.). The proteins were submitted to NFATc1 analysis by ELISA with 10 *μ*L per well (2-10 *μ*g/well) as instructed (Active Motif, CA, USA). Protein content was determined by bicinchoninic acid assay (Applygen Technologies Inc.).

For NF-*κ*B p65 analysis, nuclear protein of splenocyte (50 *μ*g/lane) from both groups at 6 h and 24 h postburn was separated by sodium dodecyl sulfate–polyacrylamide gel electrophoresis in Mini-PROTEAN® Tetra electrophoresis cell (Bio-Rad, CA, USA) and transferred onto Immobilon-P polyvinylidene difluoride (PVDF) membranes (Millipore, MA, USA) by Mini Trans-Blot® Electrophoretic Transfer Cell (Bio-Rad). PVDF membranes were incubated overnight at 4°C with the NF-*κ*B p65 antibody (1 : 1000; cat. 6956, Cell Signaling Technology, MA, USA) followed by incubation with peroxidase-conjugated secondary antibody (1 : 5000; Cell Signaling Technology, MA, USA). The blots were exposed to the super signal luminescence reagent (Applygen Technologies Inc.), and the signals were scanned by ImageQuant LAS 4000 (GE Healthcare, Germany). Then, the membranes were stripped and reprobed with lamin B1 antibody (1 : 1000; AB133741, Abcam, MA, USA) and the signals were scanned again. NF-*κ*B p65 densitometry was calculated by ImageJ software, normalized to lamin B1.

### 2.8. Cytokines, Hormones, and Biochemistry

Lung tissues 24 h after injury were emulsified with a mechanical homogenizer (Kimble Inc., NJ, USA) in a lysis buffer (0.5% Triton with 1% PMSF in 0.01 M PBS) and subjected to two rounds of freeze thaw. After being incubated at 4°C for 1 h, the homogenate was centrifuged and the supernatants were collected. The data were expressed at pg/mg protein.

Serum samples collected 24 h after injury were subjected to cytokines measurement by ELISA (ExCell Biology Inc., Shanghai, China). Serum alanine aminotransferase (ALT), aspartate transaminase (AST), urea, and creatinine were measured by commercial kits (Roche, USA) in the clinical laboratory of our hospital. Serum and plasma samples collected at 6 h and 24 h after injury were for stress hormone measurement by ELISA (Jiancheng Life Sciences, Nanjing, China).

### 2.9. Statistical Analysis

All data are expressed as group mean ± SEM. A value of *P* < 0.05 was considered significant for all analyses. The survival rate in the life table was analyzed by the Wilcoxon test. Duration of survivals has been presented as Kaplan-Meier curves with the log-rank tests comparing equality of strata. The significance of difference among groups was evaluated by the Student's *t*-test for two groups or one-way ANOVA with a post hoc LSD test for multiple comparisons when comparing three or more groups.

## 3. Results

### 3.1. Ex-4 Deteriorated Burn-Induced Mortality Independent of the SNS

**Figure 1:**
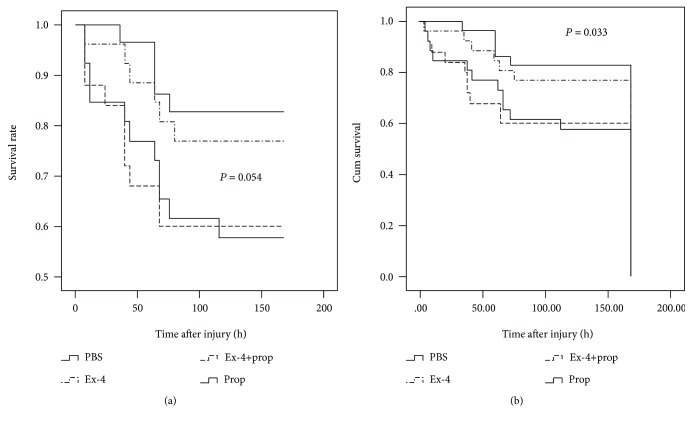
Life table of the survival rate (a) and Kaplan-Meier analysis of the survival time (b) in 15% TBSA burn-injured mice with Ex-4 treatment and its interaction with Prop pretreatment. *n* = 25 − 28 per group. *P* = 0.054 by the Wilcoxon test in survival rate and *P* = 0.033 by the log-rank test in the survival time.

The survival rate analyzed by life table revealed marginally difference in the survival rate among the groups with burn injury (Wilcoxon, *P* = 0.054, Figure 1(a)). The overall 1-week survival rate was 84.6% for single burn-injured mice (PBS), 76.9% for Ex-4-treated burn mice, 60% for Ex-4 with Prop pretreatment, and 57.7% for single Prop pretreatment. Ex-4 tended to deteriorate the survival rate of burn injury but did not reach its significance (Ex-4 vs. PBS, *P* = 0.399). Single pretreatment of Prop could significantly deteriorate the survival rate of burn injury (Prop vs. PBS, *P* = 0.021), when in combination of Ex-4, the survival rate was further worsened (Ex-4+Prop vs. PBS, *P* = 0.022).

However, there was a significant difference in the mean survival time among the groups with burn injury (log-rank test, *P* = 0.033, Figure 1(b)) as analyzed by Kaplan-Meier curves. Although the mean survival time was comparable between Ex-4-treated and vehicle mice (152.05 ± 7.59 h vs. 140.08 ± 10.44 h, *P* = 0.398), Prop pretreatment could significantly reduce burn-induced survival in PBS-treated (Prop: 115.58 ± 12.99 h, *P* = 0.020 vs. PBS) and Ex-4-treated mice (Ex-4+Prop vs. PBS: 113.54 ± 13.88 h vs. 152.05 ± 7.59 h, *P* = 0.025). Intriguingly, there was no difference of the survival time between single Prop pretreatment and that in combination with Ex-4 application (Prop vs. Ex-4+Prop: 115.58 ± 12.99 h vs. 113.54 ± 13.88 h, *P* = 0.999).

### 3.2. Ex-4 *In Vitro* Affects T Cell Function via *β*-AR, While Its Differentiation via GLP-1R

**Figure 2:**
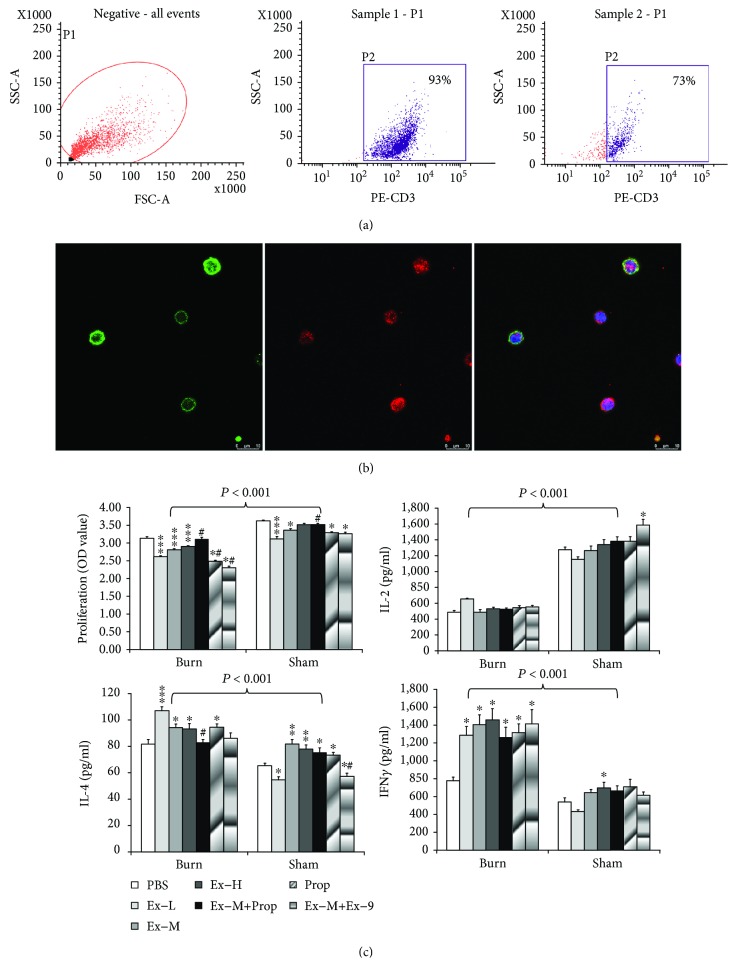
Expression of GLP-1R and its function in T cells. (a) The purified murine CD3^+^ (green) T cells were stained with rabbit anti-mouse GLP-1R antibody (red) and further detected by DyLight™ 549-conjugated goat anti-rabbit or mouse IgG. T cells incubated with control IgG did not show nonspecific binding. Nuclei are labeled with DAPI. (b) Flow cytometry of purified T cells by staining with CD3^+^ antibody. (c) T cell function and differentiation were analyzed by culturing splenocytes with ConA in the presence of PBS (ctrl), low (0.3 nmol/L, Ex-L), moderate (3 nmol/L, Ex-M), and high concentration (30 nmol/L, Ex-H) of Ex-4 with or without Prop (1 nmol/L) or exendin (9-39) (Ex-9) (1 *μ*mol/L) pretreatment. T cell proliferation and secretions of IL-2, IL-4, and IFN*γ* were presented. Data are mean ± SEM (*n* = 5/group). The differences between the groups were analyzed by one-way ANOVA. ^∗^*P* < 0.05 vs. PBS and ^#^*P* < 0.05 vs. Ex-M, by post hoc LSD analysis in one-way ANOVA or Student's *t*-test.

The purity of the adherence isolated CD3^+^ T cells was between 73% and 93%, as verified by flow cytometry (Figure 2(a)). Among these, 96% CD3^+^ T cells isolated and enriched from normal mouse splenocyte demonstrated GLP-1R-positive expression (Figure 2(b)), indicating possible direct effect of Ex-4 on T cells.

Burn injury resulted in reduced T cell proliferation and IL-2 secretion significantly (Figure 2(c)). To make things worse, Ex-4 (0.3-30 nmol/L) *in vitro* could suppress T cell proliferation in sham (*P* ≤ 0.001, one-way ANOVA) and burn mice (*P* ≤ 0.001, one-way ANOVA). Additionally, the effect of Ex-4 on T cell proliferation was dependent on *β*-AR, as Prop pretreatment could restore the inhibitory effects of Ex-M on cell proliferation in both groups. Unexpectedly, blocking GLP-1R by competitive antagonist Ex-9 could further potentiate the inhibitory effects of Ex-M on cell proliferation (Figure 2(c)), indicating that Ex-4 did not act on GLP-1R to inhibit T cell function. Nevertheless, Ex-4 could not affect IL-2 secretion significantly in splenic T cell from both groups of mice, except that Ex-9 in combination with Ex-M could moderately increase IL-2 secretion in sham mice.

Regarding T cell differentiation, burn injury could elicit a Th2-type response with higher IL-4 secretion and lower IFN*γ* secretion (Figure 2(c)). Furthermore, Ex-4 could provoke both Th1- and Th2-type responses by increasing IL-4 secretion in sham (*P* = 0.001, one-way ANOVA) and burn-injured mice (*P* ≤ 0.001, one-way ANOVA), whereas increasing IFN*γ* secretion in sham (*P* = 0.001, one-way ANOVA) and burn-injured mice (*P* = 0.003, one-way ANOVA). The increased secretions of IL-4 instead of IFN*γ* induced by Ex-4 could be reversed by Prop pretreatment in burn cells (*P* = 0.771 vs. PBS, Student's *t*-test) other than in sham cells (*P* ≤ 0.001 vs. PBS, Student's *t*-test), indicating Th2 differentiation promoted by Ex-4 might be dependent on *β*-AR. Propranolol alone could inhibit T cell proliferation mildly, but promote both IFN*γ* and IL-4 secretions from both groups significantly. On the contrary, GLP-1R blockade by Ex-9 could reverse the increased IL-4 levels by Ex-M in both groups of cells (burn and sham: *P* = 0.399 and *P* = 0.038 vs. PBS, Student's *t*-test) (Figure 2(b)). The increased IFN*γ* level by Ex-M could only be reversed by Ex-9 in cells from sham mice (*P* = 0.601 vs. PBS).

### 3.3. Ex-4 *In Vivo* Inhibited T Cell Function in Burn Mice SNS Dependently, Whereas Promoted Th2 Switch SNS Independently

**Figure 3:**
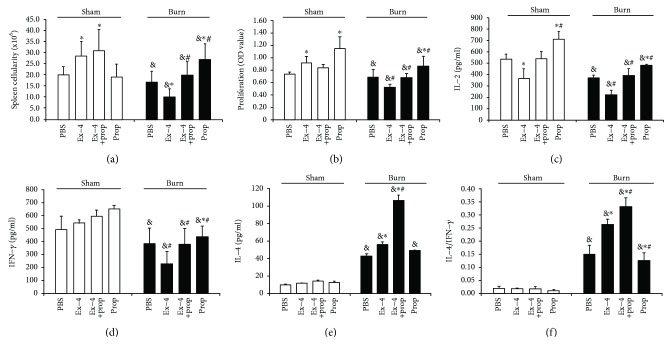
In vivo effect of Ex-4 (2.4 nmol/kg) alone or in combination with propranolol (Prop, 30 mg/kg) on splenic cellularity (a), T cell proliferation (b), IL-2 secretion (c), and differentiation (d–f) in sham and burn-injured animals 24 h after injury. Prop was administrated i.p. 30 min prior to injury, while Ex-4 was injected i.p. 30 min after injury. *n* = 5 − 8 mice/group. ^∗^*P* < 0.05 vs. PBS, ^#^*P* < 0.05 vs. Ex-4, and ^&^*P* < 0.05 vs. sham. Student's *t*-test.

Compared to the sham mice, thermal injury could result in less spleen cellularity and impaired splenic T cell function with suppressed proliferation and less IL-2 secretion (Figures 3(a)–3(c)). Consistent with the *in vitro* findings, Ex-4 *in vivo* (2.4 nmol/kg) could exert profound effect on T cell function and differentiation in both groups of mice. In detail, in sham mice, Ex-4 could mildly enhance spleen cellularity (Figure 3(a)) and T cell proliferation (Figure 3(b)), but could inhibit IL-2 secretion (Figure 3(c)), all of which could be prevented by Prop pretreatment. Even so, Ex-4 could not exert any effect on T cell differentiation (Figures 3(d)–3(f)) in sham mice *in vivo*. On the contrary, in burn mice, Ex-4 reduced spleen cellularity and suppressed T cell proliferation and IL-2 secretion which was also restored by Prop pretreatment (Figures 3(a)–3(c)). Additionally, Ex-4 promoted T cell toward Th2-type response in burn mice, which was further potentiated by Prop pretreatment (Figures 3(d)–3(f)).

### 3.4. Ex-4 Augmented Inflammatory Response in Burn Mice SNS Dependently

**Figure 4:**
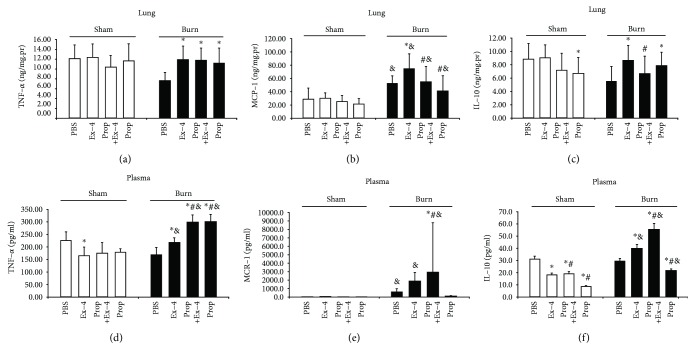
Alterations in lung (a–c) and serum (d–f) levels of TNF-*α* (a, d), MCP-1(b, e), and IL-10 (c, f) by Ex-4 (2.4 nmol/kg) with or without Prop (30 mg/kg) pretreatment in sham- and burn-injured animals 24 h after injury. Data are mean ± SEM (*n* = 8/group). ^∗^*P* < 0.05 vs. PBS, ^#^*P* < 0.05 vs. Ex-4, and ^&^*P* < 0.05 vs. sham. Student's *t*-test.

Although 15% TBSA thermal injury could not initiate higher TNF-*α* and IL-10 levels in the lung or serum, it did promote MCP-1 secretion significantly in both compartments (Figure 4). Anyway, Ex-4 exerted distinct effect on cytokine secretions between sham and burn mice. In sham mice, a bolus of Ex-4 could not exert any effect on proinflammatory cytokines in the lung 24 h following injury (Figures 4(a)–4(c)); however, it could reduce the serum TNF-*α* level at this time (Figures 4(d)–4(f)). Regarding the anti-inflammatory cytokine IL-10, Ex-4 could also reduce its level in serum rather than in the lung in sham mice, which could be restored marginally but significantly by Prop pretreatment (Figures 4(c) and 4(f)). Unexpectedly, in burn mice, Ex-4 profoundly promoted both pro- and anti-inflammatory cytokine secretions in both the lung and serum. However, the proinflammatory effect of Ex-4 was only reversed by Prop pretreatment in the lung (Figures 4(b) and 4(c)). To make things worse, they were further potentiated by Prop pretreatment in serum (Figures 4(d)–4(f)). This mixed data suggested that the induction of both the pro- or the anti-inflammatory cytokines by Ex-4 be constituent specific in burn mice and was SNS-dependent locally in organ, e.g., the lung, other than systemically in serum.

### 3.5. Ex-4 Inhibited NFATc1 Activity in CD3^+^ T Cells while Promoted NF-*κ*B Translocation in Splenocytes SNS Dependently

**Figure 5:**
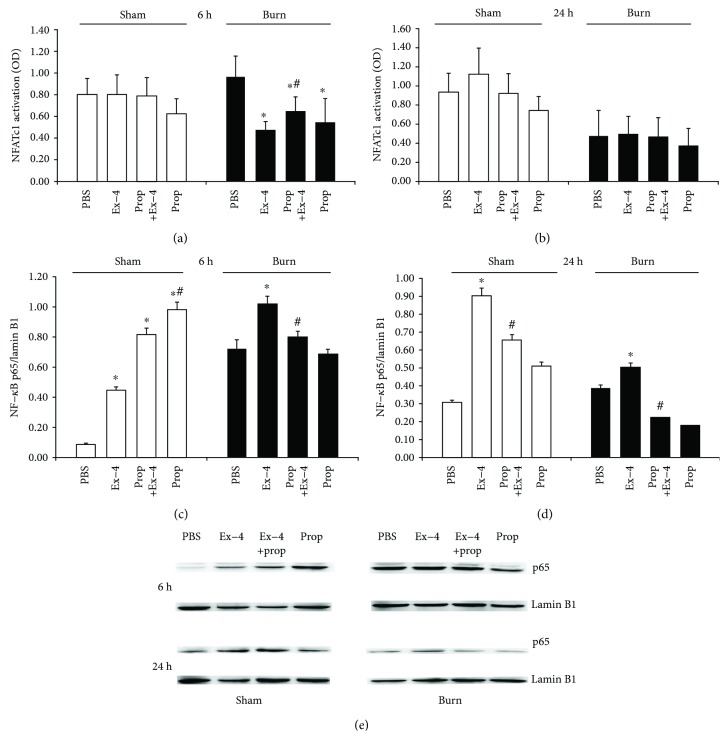
Influence of Ex-4 (2.4 nmol/kg) on nuclear NFATc1 activation in splenic T cells (a, b) and nuclear NF-*κ*B p65 translocation in splenocytes (c, d) at 6 h and 24 h following sham or burn injury with or without Prop (30 mg/kg) pretreatment. Data are mean ± SEM (*n* = 6-8 mice/group). ^∗^*P* < 0.05 vs. PBS and ^#^*P* < 0.05 vs. Ex-4. Student's *t*-test.

As opposed to that from sham mice, nuclear NFATc1 activity in purified T cells was increased in burn mice 6 h (Figure 5(a)) following injury, but was profoundly suppressed at 24 h postburn (Figure 5(b)). Although Ex-4 did not affect NFATc1 activation in T cells from sham-injured mice after 6 h *in vivo* treatment, it could significantly suppress its activity in burn-injured mice compared to that in vehicle-treated burn-injured mice. The suppression by Ex-4 could be restored by Prop pretreatment (Figure 5(a)). Over the time, the suppressed NFATc1 activity from Ex-4-treated mice was comparable to that from vehicle-treated burn mice since the NFATc1 activity was generally suppressed in burn-injured mice at 24 h postburn (Figure 5(b)).

On the other hand, nuclear NF-*κ*B p65 translocation in splenocytes as shown by Western blot (Figure 5(e)) was robustly increased in burn as opposed to sham mice 6 h following injury (Figure 5(c)) followed by a reduction to the comparable level as the sham mice 24 h postburn (Figure 5(d)). Application of Ex-4 could promote nuclear NF-*κ*B p65 translocation in splenocytes from both groups at 6 h and 24 h following injury (Figures 5(c) and 5(d)), which could be reversed by Prop pretreatment. Except that in sham T cells, the increased nuclear NF-*κ*B p65 promoted by Ex-4 was further potentiated by Prop pretreatment at 6 h postburn (Figure 5(c)).

### 3.6. Ex-4 Deteriorated the Organ Injury SNS Dependently

As expected, burn injury could profoundly augment the biomarkers for liver injury, e.g., ALT and AKT, and moderately exacerbate the biomarker for kidney injury, e.g., urea at 24 h postburn. Although Ex-4 did not do any harm to the liver in sham mice, it could raise AKT level in burn mice which could be reversed by Prop pretreatment. Regarding the ALT/AST ratio, it could be potentiated by Ex-4 SNS dependently in burn mice. In terms of kidney injury, Ex-4 could significantly raise the levels of urea and creatinine in burn rather than in sham mice. Again, the increased urea/creatinine ratio by Ex-4 was reversed by Prop pretreatment in burn mice, reinforcing the sympathetic mechanism for Ex-4 in accelerating the multiple organ syndromes in burn injury (MODS) (Table 1).

### 3.7. The Increased Serum Norepinephrine by Ex-4 Was Potentiated by Prop Pretreatment

**Figure 6:**
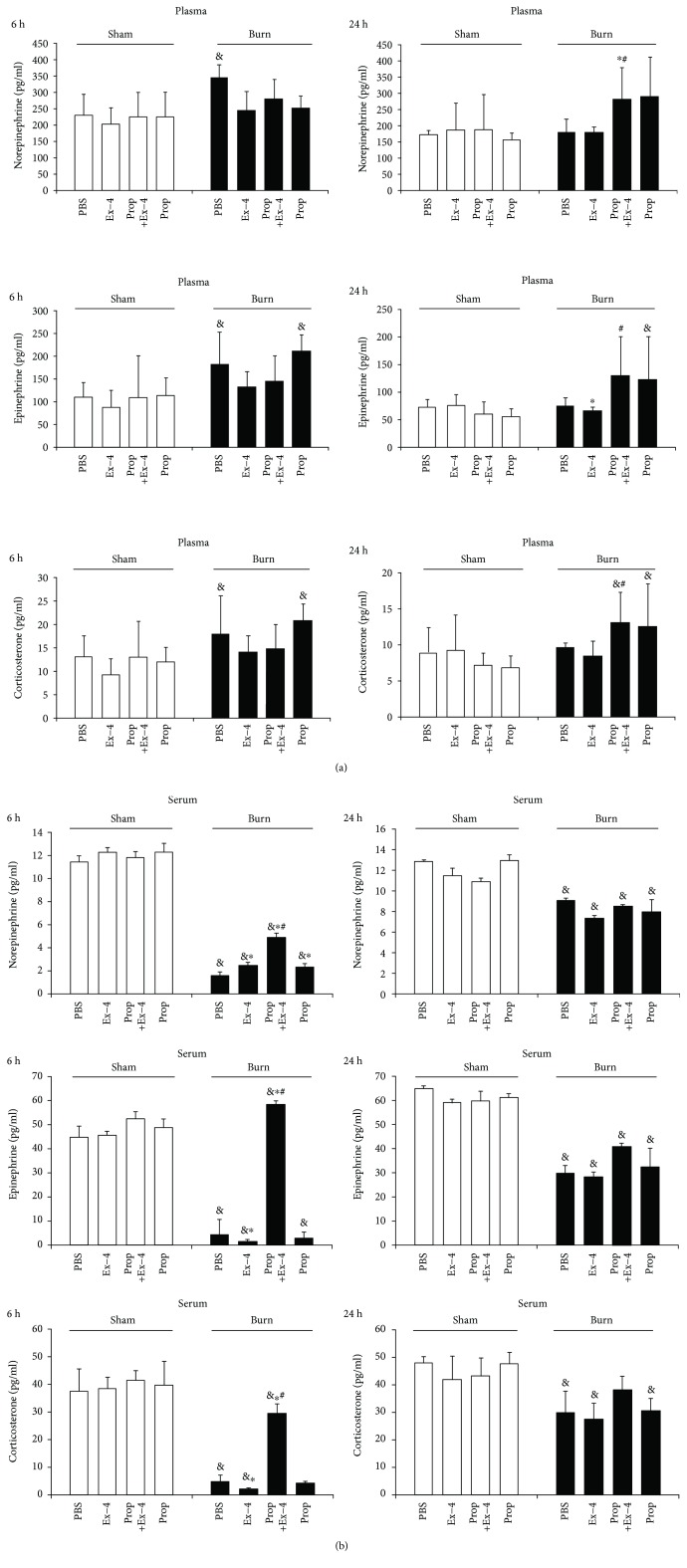
Kinetics of the effect of Ex-4 (2.4 nmol/kg) on the plasma (a) and serum (b) levels of corticosterone (A), epinephrine (B), and norepinephrine (C) in sham- or burn-injured mice that with or without Prop pretreatment. *n* = 6 − 8 mice/group. ^∗^*P* < 0.05 vs. PBS, ^#^*P* < 0.05 vs. Ex-4, and ^&^*P* < 0.05 vs. sham. Student's *t*-test.

Considering the robust activation of the SNS by Ex-4 as referred [27–29, 32], we next sought to determine the stress hormone levels in both the plasma and the serum. To our interest, the hormone profile was different between the two constituents, serum (Figure 6(a)) and plasma (Figure 6(b)). Hormone levels were ten times higher in plasma than in serum in both groups. As expected, burn injury could increase hormone levels in plasma, but neither Ex-4 nor Prop, or in combination, could affect their levels at 6 h postburn, whereas at 24 h postburn, only Ex-4 in combination with Prop could reliably increase the plasma hormone levels relative to PBS controls (Figure 6(a)).

Paradoxically, serum levels of hormones were profoundly lower in burn-injured mice compared to that in sham-injured mice at 6 h postburn (Figure 6(b)). Again in sham mice, Ex-4 exerted no significant effect on serum levels of hormones at both time points. However, 6 h after burn injury, injection of Ex-4 could significantly reduce the serum levels of corticosterone and epinephrine, whereas increase serum level of norepinephrine. Comparatively, pronounced serum levels of all the three hormones were observed in Ex-4-treated mice that with Prop pretreatment. At 24 h after injury, the decreased levels of serum hormones in burn mice gradually recovered, but were still lower than those in sham mice. At this time, a bolus of Ex-4 could not exert any effect on serum hormones in both sham and burn mice (Figure 6(b)).

## 4. Discussion

This study illustrates for the first time that modulation of the SNS by Ex-4 might deteriorate the morbidity of thermal-injured mice; however, the exacerbated mortality by Ex-4 was not dependent on the activation of the SNS by Ex-4. These data suggest that Ex-4 might regulate T cell function through two distinct pathways in burn injury. First, exogenous Ex-4 directly suppresses splenic T cell function after burn injury depending on SNS activation. Secondly, exogenous Ex-4 acts as a Th2 switch signal for splenic T cells via GLP-1R, which is further potentiated by SNS blockade. That is, Th2 switch by Ex-4 was independent on SNS activation. Removal of these two immunosuppressive functions of Ex-4 by Prop resulted in an improved T cell function with elevated NFATc1 activity, reduced splenic nuclear NF-*κ*B translocation, and consequent alleviated lung inflammation in burn-injured mice. However, Ex-4-induced Th2 switch was further potentiated by Prop pretreatment with increased systemic levels of both pre- and anti-inflammatory cytokines. Therefore, the immunosuppressive effects of Ex-4 likely override its anti-inflammatory actions in burn injury, resulting in suppressed T cell function in contrast with heightened inflammatory response in response to Ex-4 in burn injury.

We found the fluorescent GLP-1R on CD3^+^ T cell, suggesting that GLP-1 or its analogue Ex-4 could exert direct effect on T cells. As expected, Ex-4 could inhibit T cell function from sham and burn mice *in vitro* depending on *β*-AR, whereas promoted Th2 switch via the activation of GLP-1R.The distinct effects between T cell function and differentiation indicated that the two events be driven by two different pathways. Preclinical studies have provided conflicting results about the effect of Ex-4 on lymphocyte function. It was reported that Ex-4 *in vitro* could not affect the proliferation of primary thymocytes, splenocytes, lymph node cells [59], or splenic T cells from NOD mice [13]; however, it could increase the number of CD4^+^CD8^+^ T cells in lymph nodes *in vivo* [13] that leading to extensive lymphocytic infiltration [60]. Moreover, while Ex-4 (3-50 nM) *in vitro* reduced the production of cytokines from activated intestinal intraepithelial lymphocyte, acute exendin-4 administration *in vivo* robustly induced the expression of genes encoding cytokines and chemokines in the normal and injured intestine [21]. These mixed data including ours suggested that the immunomodulatory effect of Ex-4 be cell type, constituent, and immune status specific.

Considering the potency of Ex-4 in neuroendocrine activation, we went on to examine the stress hormones after Ex-4 application. Although the plasma levels of stress hormones were shortly elevated in burn as opposed to sham mice owing to the increased sympathetic tone in our study, Ex-4 could not affect the plasma levels of hormones neither in sham nor in burn mice. It has been found that GLP-1 alone could not elevate plasma catecholamine in humans or in humans during exercise [27]; however, GLP-1 in combination with 2-adrenergic antagonist yohimbine could increase plasma norepinephrine levels in humans [28]. Preclinical study also found that short-term intracerebroventricular Ex-4 could increase urinary content of catecholamine metabolites in rats, indicating a direct activation of the SNS by Ex-4 [29]. This discrepancy prompted us to further examine the levels of hormones in serum, which reflects the rapid changes of hormones. Contrary to those in plasma, the hormones in serum were ten times lower in burn mice than that in sham mice. Nonetheless, it was intriguing to find that Ex-4 could elevate the serum level of norepinephrine but could reduce those of epinephrine and corticosterone in burn mice. Additionally, Prop pretreatment could greatly elevate all hormone levels as a result of *β*-AR blockade that prevented the hormones from binding to the receptor, so the hormones were accumulated in the serum.

Then, we wonder whether Ex-4 could modulate T cell immunity via a neuroendocrine pathway. As expected, the alterations of T cell function by Ex-4 could be reversed by Prop pretreatment in both groups, indicating a SNS-dependent mechanism. In accordance, Ex-4-treated burn mice exhibited reduced cellularity of the spleen which could be restored by Prop pretreatment. It has been reported that the spleen atrophy observed in stroke may result from immune cell migration from the spleen to the cerebral injury site [52]. In our observation, the reduced spleen cellularity in Ex-4-treated burn mice indicated an increased immune cell emigration that from the spleen to the circulation, which possibly evoking an even more severe systemic inflammatory response. Moreover, studies have shown that products of the SNS, namely, epinephrine and norepinephrine, can alter the balanced expression of cytokines from Th1 in favor of Th2 *in vitro* [61]. Coinciding with the literature, the increased ratio of IL-4/IFN*γ* by Ex-4 or in combination with Prop relative to vehicle-treated burn mice was possibly due to the elevated levels of serum norepinephrine in these mice.

Actually, increased levels of cytokines from both the lung and serum were identified in Ex-4-treated as opposed to vehicle-treated mice at 24 h postburn. However, only the increased cytokines by Ex-4 in the lung other than those in the serum could be reversed by Prop pretreatment in burn mice, reinforcing an organ-exclusive SNS-dependent effect of Ex-4. To make things worse, the increased serum levels of cytokines by Ex-4 were further potentiated by Prop pretreatment, indicating a SNS-independent effect of Ex-4 in systemic immune response. It could be explained by the compartment effect of immune response that results in distinct inflammatory response among different tissues and organs, even different segments of the intestine [62]. Following the same line of evidence, splenic nuclear NF-*κ*B was consistently upregulated in Ex-4-treated burn mice compared to that in vehicle-treated burn mice depending on *β*-AR. These unexpected findings were not consistent with the consensus regarding the anti-inflammatory effect of Ex-4 *in vitro* [15] and *in vivo* [21, 22]. This paradox could be possibly attributed to the synergetic activation of the SNS by Ex-4 in burn injury. The increased serum level of norepinephrine by Ex-4 or in combination with Prop could account for the heightened systemic inflammation. Previous studies found that the neuroendocrine system activated by a 20% TBSA burn injury participates in the increased IL-6 production in the unburned skin [43]. The enhanced systemic inflammation induced by Ex-4 in burn-injured mice could be the organ- and tissue-specific differences in catecholamine stimulation, which may merely depend on regional changes in blood flow [63]. Secondly, this discrepancy could also be attributed by the difference between the acute and the chronic animal models. Consistent with our findings, acute application of Ex-4 peripherally could increase IL-6 and TNF-*α* proteins in the jejunum of mice 4 h following injection [21], and central application of Ex-4 potently increased the expressions of IL-6 and IL-1*β* mRNA in the brain 90 min following injection [64]. The third possibility could be that the outcome of *β*2-AR/NF-*κ*B interactions in the context of NF-*κ*B-driven gene expression can be either positive or negative depending on the cell type and the context [41]. Several clinical reports showed that elevated levels of catecholamine and the therapeutic *β*-blockers for severe burn injury be associated with enhanced production of inflammatory mediators [65–67] and the exacerbated hepatic inflammatory response [68] in burn patients. In our study, although TNF-*α* release was not significantly induced by 15% TBSA burn injury, MCP-1 level was greatly elevated, indicating an inflammatory response evoked. In the meanwhile, plasma stress hormone levels were greatly raised in this animal model. In this context, Ex-4 could induce higher levels of TNF-*α*, IL-10, and MCP-1 in burn mice either alone or in combination with Prop pretreatment. It is possible that the genes for these cytokines undergo synergistic expression upon coactivation of *β*2-ARs and NF-*κ*B pathway by catecholamine in the setting of burn injury.

Another two critical issues should be mentioned in our study. One is that the hormones in serum were significantly lower than that in plasma. It is asserted that serum differs from plasma only in lacking of the fibrinogen and the clotting factors [69]. The circulating steroid hormones were bound specifically to the large carrier proteins and are in equilibrium with a small fraction (less than 1%) in free solution in plasma. It is possible that the large part of the bound hormone in plasma was removed by clotting, leaving the small part of the unbound free hormone in serum. Secondly, the unbound hormones that are thought to reach the sites of biological action were further degraded [70], leading to an exhausted sympatho-adrenomedullary system following burn injury. The two events resulted in lower levels of stress hormone in serum than that in the plasma. Another issue is that Ex-4 conferred distinct effects on immune response between sham and burn mice. It could be the enhanced neuroendocrine activation in burn relative to sham-injured mice. It has been reported that neither exogenous GLP-1 nor GLP-1R antagonist altered any index of hypothalamus-pituitary-adrenal axis (HPA) axis activity in unstressed rats; however, GLP-1 could adjust HPA sensitivity to chronic stress [71]. Likewise, in our study, although Ex-4 conferred no influence on serum levels of hormone in sham mice, it could raise serum level of norepinephrine in burn-injured mice, suggesting that GLP-1 system be involved in central pathways responsible for SNS activation in burn injury.

## 5. Conclusions

In summary, we have demonstrated a novel aspect of neural suppression of immune response by Ex-4, namely, T cell function, an effect that was robust and distinct between sham- and burn-injured mice. Our results showed that a response to GLP-1R activation be mediated by the SNS that could suppress splenic T cell function accompanied by more pronounced inflammation and severe organ injuries. However, systemic inflammation was augmented by application of GLP-1R agonist, independently on SNS activation. Taken together, our findings raise important considerations for incretin-based therapy of stress or infection-induced hyperglycemia and whether those immunoregulatory effects could be related to adverse effects of drugs with GLP-1R activity.

## Figures and Tables

**Table 1:** Effects of Ex-4 on serum levels of ALT, AST, urea, and creatinine in sham- and burn-injured mice.

Groups	Treatment	ALT (U/L)	AST (U/L)	Ratio (AST/ALT)	Urea (mmol/L)	Creatinine (*μ*mol/L)	Ratio (urea/creatinine)
Sham	PBS	34.50 ± 3.50	182.00 ± 11.00	5.36 ± 0.86	11.82 ± 1.04	16.50 ± 1.50	0.72 ± 0.00
	Ex-4	37.50 ± 2.50	181.50 ± 24.50	4.91 ± 0.98	16.93 ± 0.32	26.50 ± 1.50^∗^	0.67 ± 0.13
	Ex-4+Prop	34.50 ± 1.50	197.00 ± 32.00	5.68 ± 0.68	17.01 ± 0.82	23.00 ± 0.90^#^	0.74 ± 0.04
	Prop	42.50 ± 1.50	205.00 ± 11.00	4.84 ± 0.43	14.68 ± 0.29	16.00 ± 1.00^&^	0.92 ± 0.04

Burn	PBS	291.50 ± 12.50	1689.50 ± 138.50	5.84 ± 1.07	17.08 ± 2.48	15.50 ± 2.50	1.10 ± 0.02
	Ex-4	128.50 ± 16.50^∗^	1951.00 ± 129.00^∗^	21.45 ± 3.10^∗^	24.42 ± 0.83^∗^	21.00 ± 3.00^∗^	1.18 ± 0.13^∗^
	Ex-4+Prop	169.50 ± 13.50^∗^^#^	1225.00 ± 151.00^∗^^#^	7.49 ± 2.26^#^	17.54 ± 1.37^#^	18.00 ± 1.50^#^	0.95 ± 0.14^#^
	Prop	205.00 ± 12.00^∗^	1649.50 ± 131.50^&^	9.88 ± 2.60	21.70 ± 2.14^∗^^&^	16.00 ± 2.00	1.33 ± 0.13^∗^

Animals were pretreated with normal saline or Prop 30 min prior to injury and then were injected with Ex-4 (2.4 nmol/kg) 30 min after injury. Serum was collected 24 h postburn and the biochemistry parameters were detected. Data are presented as mean ± SD. *n* = 6 − 8 mice/group. ^∗^*P* < 0.05 vs. PBS, ^#^*P* < 0.05 vs. Ex-4, and ^&^*P* < 0.05 vs. Ex-4+Prop. The values of Prop group were not compared with Ex-4 group. Student's *t*-test.

## Data Availability

The data used to support the findings of this study are available from the corresponding author upon request.

## Abbreviations

GLP-1: Glucagon-like peptide 1
Ex-4: Exendin-4
NF-*κ*B: Nuclear factor kappa B
SNS: Sympathetic nervous system
TBSA: Total body surface area
NOD: Nonobese diabetic mice
Tregs: Regulatory T cells
iNKT: Invariant natural killer T cells
MCP-1: Monocyte chemoattractant protein-1
GLP-1R: GLP-1 receptor
*β*-AR: *β*-Adrenoreceptor
Prop: Propranolol
ConA: Concanavalin A
NFATc1: Calcineurin/nuclear factor of activated T cells
Ex-9: Exendin (9-39)
DAPI: 4′,6-Diamidino-2-phenylindole
BSA: Bovine serum albumin
PVDF: Polyvinylidene difluoride
ALT: Alanine aminotransferase
AST: Aspartate transaminase
NFATc1: Calcineurin/nuclear factor of activated T cells
i.p.: Intraperitoneally
CNS: Central nervous system
MODS: Multiple organ syndrome
HPA: Hypothalamus-pituitary-adrenal axis.
